# Semantic Analysis and Topic Modelling of Web-Scrapped COVID-19 Tweet Corpora through Data Mining Methodologies

**DOI:** 10.3390/healthcare10050881

**Published:** 2022-05-10

**Authors:** Mahendra Kumar Gourisaria, Satish Chandra, Himansu Das, Sudhansu Shekhar Patra, Manoj Sahni, Ernesto Leon-Castro, Vijander Singh, Sandeep Kumar

**Affiliations:** 1 School of Computer Engineering, KIIT Deemed to be University, Bhubaneswar 751024, Odisha, India; 1829184@kiit.ac.in (S.C.); himanshufcs@kiit.ac.in (H.D.); 2 School of Computer Applications, KIIT Deemed to be University, Bhubaneswar 751024, Odisha, India; spatrafca@kiit.ac.in; 3 Department of Mathematics, Pandit Deendayal Energy University, Gandhinagar 382426, Gujarat, India; manoj.sahni@sot.pdpu.ac.in; 4 Faculty of Economics and Administrative Sciences, Universidad Católica de la Santísima Concepción, Concepción 4030000, Chile; eleon@ucsc.cl; 5 Department of Computer Science and Engineering, School of Computing and Information Technology, Manipal University Jaipur, Jaipur 303007, Rajasthan, India; vijander.singh@jaipur.manipal.edu; 6 ERCIM Postdoctoral Fellow, Department of ICT and Science, Norwegian University of Science and Technology, Ankeret, B-315, Ålesund, Torgarden, 7491 Trondheim, Norway; 7 Department of CSE, School of Engineering and Technology, CHRIST Deemed to be University, Bengaluru 560029, Karnataka, India; sandeepkumar@christuniversity.in

**Keywords:** COVID-19 sentiment analysis, BiLSTM, Latent Dirichlet Allocation (LDA), topic modeling, natural language processing

## Abstract

The evolution of the coronavirus (COVID-19) disease took a toll on the social, healthcare, economic, and psychological prosperity of human beings. In the past couple of months, many organizations, individuals, and governments have adopted Twitter to convey their sentiments on COVID-19, the lockdown, the pandemic, and hashtags. This paper aims to analyze the psychological reactions and discourse of Twitter users related to COVID-19. In this experiment, Latent Dirichlet Allocation (LDA) has been used for topic modeling. In addition, a Bidirectional Long Short-Term Memory (BiLSTM) model and various classification techniques such as random forest, support vector machine, logistic regression, naive Bayes, decision tree, logistic regression with stochastic gradient descent optimizer, and majority voting classifier have been adapted for analyzing the polarity of sentiment. The effectiveness of the aforesaid approaches along with LDA modeling has been tested, validated, and compared with several benchmark datasets and on a newly generated dataset for analysis. To achieve better results, a dual dataset approach has been incorporated to determine the frequency of positive and negative tweets and word clouds, which helps to identify the most effective model for analyzing the corpora. The experimental result shows that the BiLSTM approach outperforms the other approaches with an accuracy of 96.7%.

## 1. Introduction

The micro-blogging and social networking site Twitter exhibits a leading platform for several individuals and organizations for expressing their views and opinions, sharing their thoughts, and keeping them up to date with day-to-day social and political affairs [1]. Twitter has about 145 million day-to-day active users and 330 million monthly active users, making it an important source for gathering tweets for research [2]. Twitter had a restriction of 140 characters, but in the year 2017 Twitter doubled the character count to 280 characters for every tweet, which compels users to adapt phrases in their tweets [3]. Twitter has over 1 billion unique tweets posted every day and obtains 15 billion API calls every day [1]. Twitter sentiment analysis aims at determining sentiment polarity (tweets are positive or negative [2]). 

In December 2019, a list of patients with pneumonia of an unidentified cause was first reported in Wuhan, China. It was found that the patients were linked to a wet animal and seafood wholesale market in Wuhan [4]. COVID-19 is caused by a novel human pathogen that developed from bats and ultimately jumped to the human being via an intermediary host [5]. The outbreak of COVID-19 spread all over the world at an exponential rate. The disease spreads through human contact and tiny droplets formed when a person sneezes, coughs, or talks. It has symptoms like cough, fever, diarrhea, and shortness of breath, and in harsh cases, it can also cause pneumonia and sometimes even death. COVID-19 has an incubation period of more than two weeks [6]. On 11 March 2020, the World Health Organisation (WHO) declared this rapidly growing disease, COVID-19, a pandemic [7]. By 16 October, the WHO had reported 38,789,204 confirmed COVID-19 cases and 1,095,097 deaths globally and 7,370,468 confirmed cases of COVID-19 in India with 112,161 deaths [8]. Amidst the ongoing coronavirus (COVID-19) pandemic, the entire world is witnessing a paradigm shift in day-to-day activities, be it learning online or the way we interact, socialize, do shopping, or conduct business.

By the first week of March 2020, many nations like China, Spain, Italy, and Australia were combating the pandemic by taking rigorous measures by blocking the areas having a risk of community transmission and via a nationwide lockdown. Taking suggestions from these foreign countries, the Indian government made a crucial decision for a nationwide lockdown of 21 days from 26 March to 14 April 2020 and then extended this until 31 May 2020 [9]. A country with 1.3 billion people was at an immense risk of suffering from inevitable destruction, and therefore, harsh measures were expected. For a novel coronavirus without any accessible vaccine or therapeutic drug, one of the strategies may be community mitigation, which consists of social distancing along with the closing of schools, colleges, restaurants, bars, movie theatres and practicing work from home. Social gatherings like marriage, graduations, festivals, and sports events were canceled or discouraged [10]. Isolation and change in lifestyle are linked with depression, stress, fear, and post-traumatic stress disorder (PTSD) and may also lead to lost social and family supports [11]. To stay up-to-date, individuals used social media. The leading problem remained the same about the viral spread, immunity, post recovery, drug therapy, and vaccine, so many people moved to social networking sites for resolution where they found lots of pandemics-linked discussion about school shutdown, economy, absence of medical supplies, and withdrawing social associations. With the spread of COVID-19 infection globally, activity on social networking sites such as Twitter, Facebook, Instagram, and YouTube began to expand [12]. Millions of people took to Twitter for sharing their views, ideas, opinions, and reactions due to this extreme crisis. 

This paper focuses on analyzing the tweets of users during this nationwide lockdown and pandemic, i.e., whether they were tweeting positively or negatively. Due to the short length of tweets, it is a bit challenging to perform sentiment analysis on the Twitter dataset. As the data were disordered and composite, data pre-processing, which includes URLs removal, replacing negation, removing numbers, stop words removal, and expanding acronyms, was done before the feature extraction step. We used Natural Language Tool Kit (NLTK) [13] to process the data gathered from the dataset, which is further discussed in the experimental section. The Twitter-specific features were eliminated to form normal text, and then feature extraction was performed to extract more features.

We highlight our objectives behind the study and the motivation for developing a new model which can help in analyzing semantic texts. The analysis of the tweets is very useful in determining the sentiments of the people, whether the people are tweeting positively or negatively. Tweets have wide impact on the public temperament; therefore, it is very important to know the polarity of the tweets. A negative tweet can be filtered out. In addition, we can keep track of negative tweets and provide assistance if required. Thus, we can also track a person who continuously tweets negatively. Our main motivation behind the study is to provide a comparison between various algorithms and provide the best model in terms of accuracy and other result parameters, which are further discussed in the experimental section. Our second main goal was to provide our own mined dataset which can be used in further studies and contribute to the research society. This dataset is very useful and necessary as it has been mined from Twitter and the sentiments were found using the best algorithm after comparison of eight different models. We explain the methods that were used for collecting the data samples and pre-processing steps. We also provide the results of the best model that was stated from the previous dataset and apply them to our newly created dataset for more accurate analysis and efficiency. The main contributions of the paper are jotted down in the following points:

- Our own mined COVID-19 dataset from Twitter API is proposed, consisting of 6648 tweets.
- Our mined dataset has been compared with the other two trained datasets.
- Topic modeling with the help of LDA has been performed on all datasets.
- RNN network, BiLSTM, and various other classification algorithms have been performed, and the ROC curve has been found for all of these to select the best among them.

The remaining part of the paper has been arranged into many sections. Section 2 briefs about works related to COVID-19 semantic analysis. Section 3 describes the methodology and materials which explain the statistics inspection, data pre-processing, and feature extraction. Section 4 describes the topic modeling technique, Latent Dirichlet Allocation (LDA), the Bidirectional Long Short-Term Memory (BiLSTM) algorithm, and various algorithms implemented in the paper, namely, support vector machine, naïve Bayes [14], logistic regression-stochastic gradient descent, logistic regression, decision tree, random forest [15] and Majority Voting Classifier (MVC). Section 5 comprises of scrutiny, results, and comparison of models, followed by Section 6, which discusses the analysis and discussion of the results obtained in the experiment. Section 7 constitutes the conclusion and future work. 

## 2. Related Work

COVID-19 has evolved as one of the major challenges in the world due to its highly mutating, contagious nature. Tweets have a wide impact on public emotions; therefore, it very important to know the polarity of tweets. In this paper we review several articles related to sentiment analysis from COVID-19 tweets collected from a Kaggle dataset using various deep learning and machine learning models.

Hung et al. (2020) [10] applied Natural language Processing (NLP), a Machine Learning (ML) technique for analyzing and exploring the sentiments of Twitter users during the COVID-19 crisis. The hidden semantic features in the posts were extracted via topic modeling using Latent Dirichlet Allocation (LDA). Their dataset was originated exclusively from the United States and tweeted in English from 20 March to 19 April 2020. They analyzed 902,138 tweets, out of which semantic analysis classified 434,254 (48.2%) as positive, 280,842 (31.1%) as negative, and 187,042 (20.7%) as neutral. Tennessee, Vermont, Utah, North Dakota, North Carolina, and Colorado expressed the most positive sentiment, while Wyoming, Alaska, Pennsylvanian, Florida, and New Mexico conveyed the most negative tweets. The themes that were considered in the experimental section included health care environment, business economy dominant topic, social change, emotional support, and psychological stress. However, the authors do not provide any industrial level model that can be implemented for analyzing these themes and provide conclusive results, unlike our experiments where models can provide different results based upon the tone, speech, etc. of the text given. Xue et al. (2020) [16] also applied the Latent Dirichlet Allocation (LDA) technique for topic modeling and identified themes, patterns, and structures using a Twitter dataset containing 1.9 million tweets associated with coronavirus gathered from 23 January to 7 March 2020. They identified 10 themes including “COVID-19 related deaths”, “updates about confirmed cases”, “early signs of the outbreak in New York”, “cases outside China (worldwide)”, “preventive measures”, “Diamond Princess cruise”, “supply chain”, “economic impact”, and “authorities”. These results do not reveal symptoms and treatment-related messages. They also noticed that panic for the mysterious nature of COVID-19 prevailed in all themes. Although the study talks about the procedure used in the experiment comprising of machine learning techniques, the study does not provide any experimental results or analysis which can be used as a model. In comparison, in our work we have used machine learning techniques and have experimented with our best models that have been tested on two Kaggle datasets and got the result. This result has been compared with our own mined dataset (the dataset was mined from Twitter using the keyword COVID-19 and generated 6648 tweets). We have also attached the label for every tweet.

Muthusami et al. (2020) [17] aimed to inspect and visualize the impact of the COVID-19 outbreak in the world using Machine Learning (ML) algorithms on tweets extracted from Twitter. They utilized various machine learning algorithms such as naïve Bayes, decision tree, SVM, max entropy, random forest, and LogitBoost for classifying the tweets as positive, neutral, and negative. LogitBoost ensemble classifier with three classes performed better with an accuracy of 74%. However, authors lack in terms of their model’s accuracy when compared to our models used in the different datasets. Similar work was presented by Lwin et al. (2020) [18] investigating four emotions, namely, anger, fear, sadness, and joy, during the COVID-19 pandemic. They collected 20,325,929 tweets from Twitter during the initial phase of COVID-19 from 28 January to 9 April 2020 using the keywords “Wuhan”, “corona”, “nCov” and “COVID”. They found that social emotions altered from fear to anger throughout the COVID-19 crisis, while joy and sadness also surfaced. Sadness was indicated by topics of losing family members and friends, while gratitude and good health showed joy. 

Chakraborty et al. (2020) [19] analyzed the kinds of tweets collected during this COVID-19 crisis. The first dataset containing 23,000 tweeted posts from 1 January 2020 to 23 March 2020 had a maximum number of negative sentiments while the second dataset contains 226,668 tweets collected from December 2020 to May 2021, which contrasts the greatest number of negative and positive tweets. They utilized bag-of-words vectorizers like TF-IDF vectorizer and count vectorizer from the sklearn library for word embedding purposes. They used various classifiers such as ensemble models, naïve Bayes models, Bernoulli classifier, multinomial classifier, support vector machine models, AdaBoost, logistic regression, and LinearSVC. The best classifier was naïve Bayes with an accuracy of 81%. Li et al. (2020) [20] analyzed the effect of COVID-19 on the psychological well-being of people by organizing different trials on sentiment analysis using microblogging sites. It was established that information gaps in the short-term in individuals change with psychological burdens after the outbreak. They used Online Ecological Recognition (OER), which automatically recognizes psychological conditions such as anxiety, well-being, etc. of a person. Bakur et al. (2020) [21] studied the sentiments of Indian people post lockdown enforced by the Indian government. They collected about 24,000 tweets obtained from the handles #IndiafightsCorona and #IndiaLockdown in the period of 25 to 28 March 2020. The study was concluded only by using Word cloud and the study depicts that Indians took the lockdown decision positively.

Imran et al. (2020) [22] used deep learning models like Long Short-Term Memory (LSTM) to analyze tweets related to the COVID-19 crisis. They utilized different datasets such as the Sentiment140 dataset containing 1.6 million tweets, an emotional tweet dataset, and a trending dataset on COVID-19. For comparison, they also trained Bidirectional Encoder Representations from Transformers (BERT), GloVe, BiLSTM, and GRU. Wang et al. (2020) [23] fine-tuned the Bidirectional Encoder Representations of Transformer (BERT) model for classifying the sentiments of Chinese Weibo posts about COVID-19 into positive, negative, and neutral and analyzed the trends. The dataset contains 999,978 tweets from 1 January 2020 to 18 February 2020. The model achieved an accuracy of 75.65%, which surpasses many NLP baseline algorithms. However, the accuracy is lacking when compared to our results.

Sitaula et al. (2021) [24] conducted an analysis on COVID-19 tweets in the Nepali language. They utilized different extraction methods such as domain-agnostic (da), domain-specific (ds), and fastText-based (ft). They also proposed three CNN methods and ensembled three CNN methods using CNN ensemble. They made a Nepali Twitter sentiment analysis dataset. Their feature extraction technique has the capability to discriminate characteristics for sentiment analysis. Shahi et al. (2022) [25] demonstrated text representation methods fastText and TF-IDF and a combination of both to gain hybrid features. They used nine classifiers on NepCov19Tweets, which is a dataset of COVID-19 tweets in the Nepali language. The best classifier was SVM with a kernel Radial Bias Function (RBF) with an overall classification accuracy of 72.1%. Sitaula et al. (2022) [26] combined the semantic information generated from the combination of the domain-specific (ds) fastText-based (ft) methods. They used a Multi-Channel Convolutional Neural Network (MCNN) for classification purposes. They found that the hybrid feature extraction technique performed better with 69.7% accuracy, while the MCNN also performed much better than an ordinary CNN with 71.3% accuracy.

The above-presented studies which we included in this section cover various themes and other analysis of the sentiments but lack the provision of any machine-learning-based model which can help in doing the same with other tweets or messages. However, out of nine studies shown above, only two studies presented a model-based application. Furthermore, these models lack in terms of accuracy when compared to our experimental models. Apart from the models, previous studies lack in comparing their outcomes with other datasets to have a deeper insight into the sentiments of the tweets. We, in our experiment, include a new approach in which we first try different models on the previously collected datasets (varying in size), and after getting the model, we introduce our new dataset collected based upon the understandings and algorithms. We also check the best model on our dataset to check how varied the results are and how they can improve the work. Table 1 provides a summary of the dataset.

## 3. Materials and Methods

**Figure 1:**
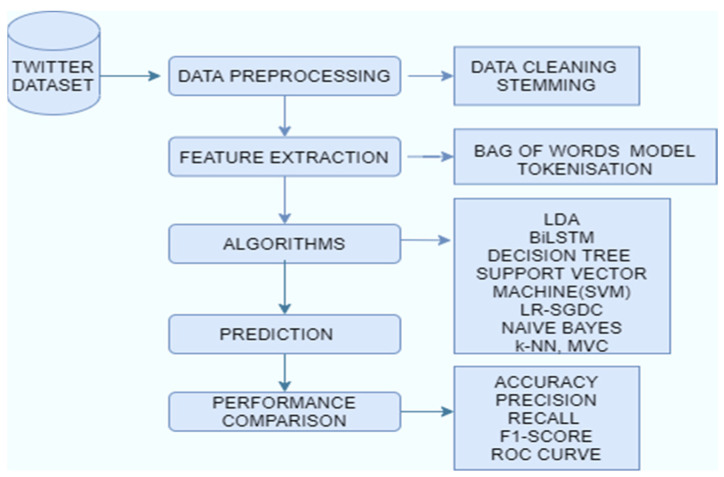
Workflow of COVID-19 sentiment analysis.

Machine learning is a trending technology where algorithms enhance automatically by automatically learning the relationships found in data [27]. This research paper deals with classifying the tweets related to COVID-19 into positive or negative sentiments. The dataset was pre-processed before applying it to the model. The pre-processing steps include stopwords removal, stemming, and tokenization. Latent Dirichlet Allocation (LDA) was done for topic modeling. Data classification techniques like BiLSTM [28], random forest, naïve Bayes, LR-SGD classifiers, logistic regression, decision tree, SVM, and MVC were used to categorize the tweets into positive or negative. These methods were used as these are promising classifiers and techniques for analyzing the polarity of tweets. These classifiers have been successfully applied in many applications such as social media text analysis, emotion analysis, text analysis, etc. These technologies also prove beneficial in analyzing the thought processes of the general public. These approaches can successfully classify and express the opinion and feelings of human beings. LDA for topic modelling will be very much beneficial in recognizing the pattern of the tweets. We can find a group of words which are mainly involved in the negative or positive tweets. Later on, we can select those tweets which contain these types of words. The count vectorization and tokenization technique provide a vector for experimentation of the models and are a very well-known feature extracting technique. Figure 1 shows the workflow of the COVID-19 sentiment analysis. For the experimental workflow, we used the Keras library (using TensorFlow backend) on an anaconda environment. All models were trained through Python 3 using high-level APIs for the construction of neural networks in the Bi-LSTM model. We used an i5 8th generation processor with a 16GB RAM.

### 3.1. Data Exploration

In this research paper, three different datasets were taken. The first dataset [29] was taken from Kaggle in CSV format consisting of 648,958 tweets with 177,456 unique tweets, and the remaining 471,412 tweets were retweeted by the users. As the retweeted posts contain the same tweets and sentiments, we removed those tweets. These tweets were related to COVID-19 and the sentiments of people in India during lockdown from 20 March to 31 May 2020.

**Figure 2:**
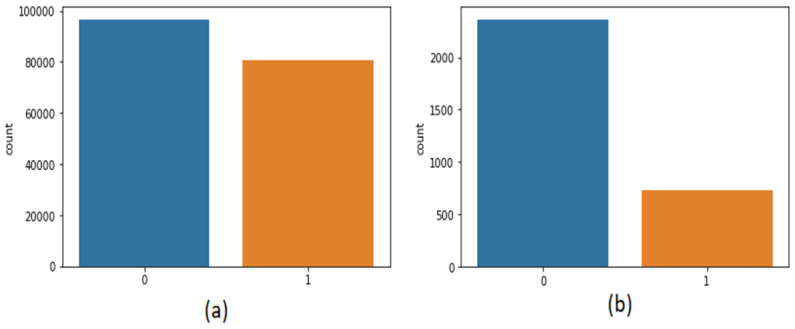
Frequency of positive or negative dataset of (**a**) first dataset (**b**) second dataset.

**Figure 3:**
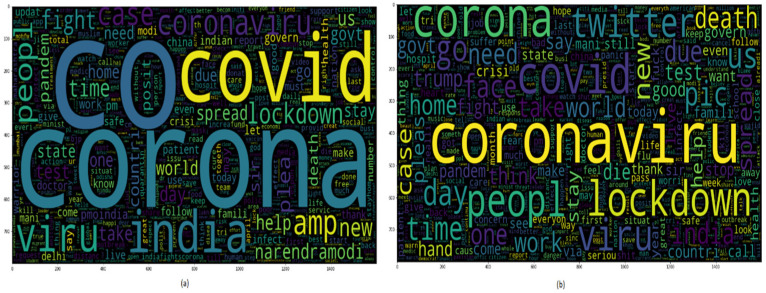
Word cloud of the dataset.

The second dataset [30] was also taken from Kaggle, which contains 3090 tweets related to the coronavirus and lockdown in India from 23 March to 15 July. The third dataset is self-mined tweets from Twitter API containing 6648 tweets. Inspecting the data has an important part in machine learning as it assists us in imagining the class and statistics of corpora. Figure 2 shows the statistics of positive or negative tweets of both the datasets. As it is a textual dataset, the word cloud can also be seen in Figure 3.

### 3.2. Data Preprocessing

The dataset contains ill-formed words, heterogeneous, unstructured, non-dictionary terms, and irregular grammar, so before the feature extraction step, the tweets were cleaned using the numerous NLTK techniques [2]. The various pre-processing steps are [31]:

- Removing non-ASCII and non-English characters from the text.
- Eliminating the HTML tags and URL links.
- Removing numbers and extra white spaces, as they do not impart any facts about sentiment.
- Removing the special characters such as @, $, *, #, etc.
- Converting all the letters into a smaller case.
- Eliminating English literature stopwords such as “an”, “about”, “as”, “any”, etc., as these words are not involved in detecting the polarity of sentiments.
- Stemming was done to bring back the word to its root form such as “strength” becomes “strong”, “better” becomes “good”, and so on.

### 3.3. Feature Extraction

Feature extraction is the premier step of Natural Language Processing (NLP). The text data cannot be fed directly in its original form into the machine learning or deep learning models so these words are encoded into numbers and these numbers are represented as vectors.

#### 3.3.1. Count Vectorizing

This is a basic encoding technique where a vector of size equal to the size of the English dictionary is taken with all its elements initialized to zero. Every time text data points to a vocab word then the element in the vector representing that word will be increased by one “1”, leaving zeroes in each place of the vector where the word was not found even once, as shown in Equations (1) and (2). A vector was created with 171,476 words of the Oxford English Dictionary [32], and so the architecture will have high feature selection, and thus, high variance is noted. Here, the count vectorizer keeps track of the rare as well as the most frequent words of the corpora. Feature extraction is the dimensionality reduction technique used for eliminating rare and non-informative words. A bag-of-words model consisting of the 1500 most frequent words of the corpora is created from the feature vector to enhance the accuracy of the model [2].

(1) Day=(0, 0, 0, 1, 0, 0, 0…0)

(2) Time=(0, 0, 0, 2, 0, 0…0)

#### 3.3.2. Tokenization

Breaking up raw text into unique text, i.e., tokens, is known as tokenization. Every token has different token ids. In tokenization, a vector of size equal to the corpora is created. A token sequence is created and represented as a vector, as demonstrated in Equations (3) and (4). Due to the difference in length of each tweet and its corresponding vector sequence, it is very tough to feed in deep learning models as it needs sequences of equal length [33]. This issue has been countered using truncating and padding steps. If the tokenized sequence length is greater than the padded sequence length, then the extra length needs to be truncated, and if the length is smaller than the sequence, it is padded with ‘0’. On choosing the sequence length to be 6, then truncating will happen to Equation (3) and padding to Equation (4), as shown in Equations (5) and (6), respectively.

(3) One should stay calm in difficult times=(322, 134, 21, 12, 78, 456, 563)

(4) Practice makes a man perfect=(536, 32, 312, 41, 444)

(5) One should stay calm in difficult times=(322, 134, 21, 12, 78, 456)

(6) Practice makes a man perfect=(536, 32, 312, 41, 444, 0)

## 4. Algorithms

One of the most crucial parts of supervised machine learning is the classification algorithms which find the class of the data. This research paper utilizes various classification algorithms for classifying the tweets as positive or negative. 

### 4.1. Latent Dirichlet Allocation (LDA)

**Figure 4:**
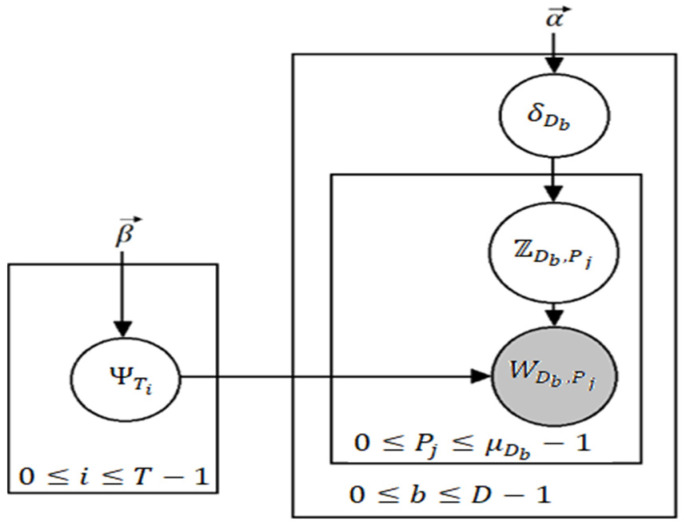
Plate notation for LDA where grey signifies an entity that can be observed.

In the context of topic modeling, Latent Dirichlet Allocation (LDA) [34] is the most famous in terms of popularity and its usability. It is a generative model [35] used for topic modeling; however, it is more widely known as a dimensionality reduction technique. Topic modeling can be defined by the process in which a machine predicts the most pertinent and relevant topics in an input corpus. Now, we explain how LDA achieves this. A general assumption is made by LDA that there is a vocabulary having P indistinguishable words and T different topics where every word can be represented as $P_{j}$ such that 0 ≤ *j* ≤ *P* − 1. Similarly, each topic represented as $T_{i}$ (0 ≤ *i* ≤ *T* − 1) represents a probability distribution $\Psi_{T_{i}}$ over P words, each having a Dirichlet prior β. Now, $\Psi_{T_{i},P_{j}}$ is the probability that the word $P_{j}$ represents the topic $T_{i}$. Having a total of D documents (here, documents do not mean full instances of articles or reports, but a small block of text such as a paragraph), then we can say β yields the distribution of *T* topics over *D* documents. If we take a variable *Z* denoting the assignment of topics to every word, then a document can be considered to have a mixture of different topics. We assume there are $\mu_{D_{b}}$ words in a document $D_{b}$ (0 ≤ *b* ≤ *D* − 1) and that $\delta_{D_{b}}$ is the probability distribution of documents over the topics drawn from Dirichlet distribution parameterized by α. Figure 4 denotes the plate notation for LDA.

Clearly, $\delta_{D_{b},T_{i}}$ is the probability that $D_{b}$ is associated with $T_{i}$. For now, we assume that α and β are scalars (in Figure 1 and defining the Dirichlet distribution we take them to be vectors, however); LDA iterates through all the documents $D_{b}$ which have $\mu_{D_{b}}$ words. For every word $P_{j}$ a topic assignment is drawn from $Z_{D_{b},P_{j}}$ from the categorical distribution $\delta_{D_{b}}$, after which a word $W_{D_{b},P_{j}}$ is drawn from a categorical distribution $\Psi_{Z_{D_{b},P_{j}}}$. The following are the steps of the algorithm:

1. Draw $\delta_{T_{i}}\;\sim \;Dirichlet\left(\beta \right)$ for each 0≤i≤T−1.
2. Consider $D_{b}$ for each 0≤b≤D−1
  - Draw $\Psi_{T}\;\sim \;Dirichlet\left(\alpha \right)$
  - Draw $Z_{D_{b},P_{j}}\;\sim \;Ca\left(\delta_{D_{b}}\right)$ for each 0≤b≤D−1
  - Draw $W_{D_{b},P_{j}}\;\sim \;Ca\left(\Psi_{Z_{D_{b},P_{j}}}\right)$ for each 0≤b≤D−1.

Ca is the distribution of the categories with the Dirichlet distribution denoted as Dirichlet having arguments or Dirichlet priors as α or β. If we take vector, Dirichlet is given in Equation (7).

(7) $$Dirichlet\left(\delta_{D_{b}}|\alpha \right)=\frac{1}{\mu \left(\alpha \right)}\prod_{b=0}^{D-1}D_{b}^{\alpha_{b}-1},$$

where μ is the Beta distribution. It is defined having input α in Equation (8).

(8) $$\mu \left(\alpha \right)=\frac{\prod_{b=0}^{D-1}\tau \left(\alpha_{b}\right)}{\tau (\sum_{b=0}^{D-1}\alpha_{b})},\;\alpha =\left(\alpha_{0},\;\alpha_{1},\;\alpha_{2},\;\ldots ,\;\alpha_{D-1}\right)$$

τ(y) in Equation (8) is given in Equation (9).

(9) τ(y)=(y−1)!

We remark that τ(y) is more popularly known as the complete gamma function. LDA is used in plenty of applications including web-spam filtering [36], tag recommendation [37], bug localization [38], etc. LDA has also been used for annotation of satellite images to segment different types of regions such as golf courses, deserts, urban areas, etc. [39].

### 4.2. Bidirectional Long Short-Term Memory

**Figure 5:**
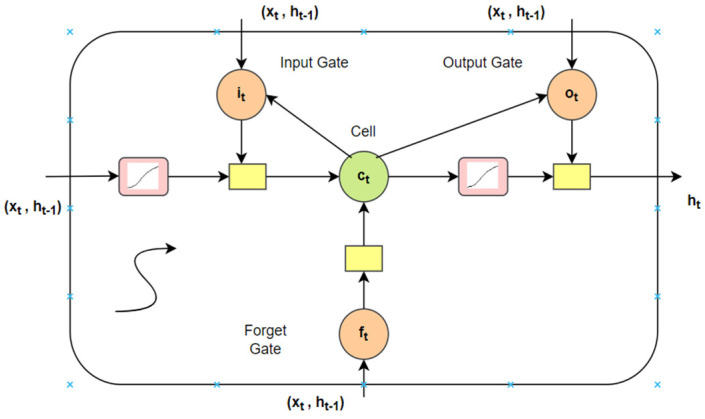
LSTM memory cell.

A conventional neural architecture cannot recall the prior inputs but a Recurrent Neural Network (RNN) has the ability to memorize and recall due to the loops and hidden layers in between them. A RNN converts the independent activations to reliant activations by appointing the same weights and biases to complete the layers, and the outcome of a layer is input to the next hidden layer. LSTM is a particular form of RNN which abstains from the extensive dependencies [40]. The long short-term memory cell stores the hidden layer of a RNN. The memory cell if LSTM can be attained via Equations (10)–(14). Figure 5 denotes the LSTM memory cell.

(10) $$i_{t}=\sigma \left(W_{xi}x_{t}+W_{hi}h_{t-1}+W_{ci}c_{t-1}+b_{i}\right)$$

(11) $$o_{t}=\sigma \left(W_{xo}x_{t}+W_{ho}h_{t-1}+W_{co}c_{t-1}+b_{o}\right)$$

(12) $$f_{t}=\sigma \left(W_{xf}x_{t}+W_{hf}h_{t-1}+W_{cf}c_{t-1}+b_{f}\right)$$

(13) $$c_{t}=f_{t}c_{t-1}+i_{t}tanh\left(W_{xc}x_{t}+W_{hc}h_{t-1}+b_{c}\right)$$

(14) $$h_{t}=o_{t}\tanh\left(c_{t}\right)$$

where logistic sigmoid function is represented by σ. The forget, cell vectors, output and input gate are represented by *f*, *c*, *o*, and *i*. The dimensions of these are the same as of the hidden vector *h* [40].

**Figure 6:**
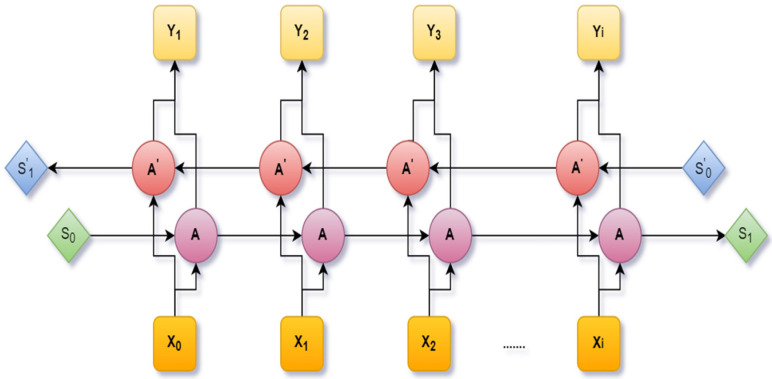
A bidirectional LSTM network.

An extension of the LSTM is the Bidirectional Long Short-Term Memory (BiLSTM), which was designed by including two independent LSTM cells. A fixed sequence to sequence problem was solved using Bi-LSTM. It is very much efficient in a text dataset where the input has various lengths. Through this architecture, the neural network can have both backward and forward details at every time interval. Figure 6 shows the Bi-LSTM [41].

### 4.3. Logistic Regression

Logistic Regression (LR) is among those analytical or mathematical methods that have been demonstrated to be highly authentic while performing sentiment analysis. It is a lot easier to interpret, implement, and train efficiently. It is much less inclined towards overfitting. In high-dimensional data overfitting can happen, but that can be avoided by using L1 and L2 regularization. The independent variables in this algorithm are observed as the predictor of the dependent variable. It is less prone to overfitting in a low-dimensional dataset. It proves very efficient when the dataset has features that are linearly separable. The relation between the independent and dependent variables is nonlinear and can be treated as a particular instance of a common linear model. It has a binomial distribution in place of Gaussian distribution since the dependent variable is categorical [42,43]. It gives back the probability by converting the result with the assistance of the logistic sigmoid function. If the predicted value is greater than 0.5, then it is marked as positive or else negative. Nonlinear problems cannot be solved using logistic regression. It is also difficult to capture a complex relationship. The linear regression equation is specified in Equation (15).

(15) $$Y=C_{0}+C_{1}X$$

The equation of sigmoid function is given in Equation (16).

(16) $$S=\frac{1}{1+e^{-y}}$$

Now, putting Equation (16) to Equation (15) and analyzing for y, we get Equation (17), i.e., logistic regression computation.

(17) $$\ln\left(\frac{S}{1-S}\right)=C_{0}+C_{1}X$$

### 4.4. Support Vector Machine 

A Support Vector Machine (SVM) is a well-known ML method for maximizing the predictive result by automatically avoiding the overfit to the data by building a decision line between the two classes, i.e., positive or negative [44]. A SVM is very efficient in high dimensional databases. A SVM is very capable of delivering capable results due to complex complexity problem. It is effective in cases where the number of dimensions is greater than the number of samples. A SVM does not perform very well on noisy data. It will underperform where the number of features for each data point exceeds the number of training data samples. The decision line also known as the hyperplane is aligned such that it is far away from the nearest data points from each of the categories. A SVM detects the hyperplane by calculating the Euclidean distance between two data points. These nearest points are known as support vectors. The distance between two support vectors is called a margin. The margin of the hyperplane [45] can be calculated by using Equation (18).

(18) $$margin\equiv arg_{x\in d}\min D\left(x\right)=arg_{x\in d}\min\frac{\left|x\times w+b\right|}{\sqrt{\sum_{j=1}^{D}w_{j}^{2}}}$$

A SVM aims to identify the class correctly so the mathematical calculations of the SVM are given in Equations (19) and (20).

(19) $$wx_{j}+b\geq 1;\;\;\;for\;y_{j}=1$$

(20) $$wx_{j}+b\leq 1;\;\;\;for\;y_{j}=-1$$

The optimal hyperplane can be defined in Equation (21).

(21) $$wx_{j}+b=0$$

where $x_{j}$ represents the feature vector, w refers to the weight vector, and *b* is the bias. SVMs are implemented using kernels. Here, a linear SVM kernel is used whose mathematical equation is defined in Equation (22).

(22) K(x,y)=sum(x∗y)

### 4.5. Naïve Bayes

The naïve Bayes classification algorithm is the most ordinary supervised machine learning model which evaluates the probability of a current observation belonging to a predetermined class, using a Bayes’ theorem with naïve independence presumption between the features [46]. Naïve Bayes is an elementary technology for classifiers construction. This algorithm does not require much training data and is highly scalable with numerous data points and predictors. Real-time predictions can be attained very easily due to its fast-implementing nature. A naïve Bayes algorithm makes use of bag-of-words features to recognize the sentiments of tweets. They perform the classification work by correlating the use of tokens with positive or negative tweets and then by using the Baye’s theorem to estimate the probability that the tweet is a positive tweet or not. A multiclass prediction problem can easily be solved using this classifier. In the case of a categorical input variable, this classifier performs the best. The technology determines the previous probability of each class based on the training set and presumes that classification could be predicted by considering the posterior probability and conditional density function [42]. The posterior probability can be evaluated using Equation (23). The main drawback is that it assumes all the features are independent, and in real life, it is very hard to find a set of independent features.

(23) $$P\left(N_{j}|V\right)=\frac{P\left(N_{j}\right)P\left(V|N_{j}\right)}{P\left(V\right)}$$

where $P\left(N_{j}|V\right)$ is the posterior probability; $P\left(V|N_{j}\right)$ presents the chance, i.e., the probability of V when $N_{j}$ is true; *P*($N_{j}$) is the prior, i.e., the possibility of $N_{j}$; and *P*(*V*) presents the marginalization, i.e., the probability of *V*.

As the training data are independent and all of them contribute equally to the classification problem, a simple method of naïve Bayes has been developed. Due to the conditional independence, $P\left(V|N_{j}\right)$ could be evaluated using Equation (24).

(24) $$P\left(V|N_{j}\right)=\prod_{i=1}^{k}P\left(V_{i}|N_{j}\right)$$

The prediction is made for the category with the greatest posterior probability [42], given in Equation (25),

(25) $$S_{j}=\mathrm{argmax}P\left(S_{j}\right)\prod_{i=1}^{k}P\left(V_{i}|S_{j}\right)$$

where j={positive,negative}.

### 4.6. Decision Tree

Decision Tree [47] categorizes the leveled trained data into rules or trees [48]. It is a technique for approximating discrete-valued functions that is powerful with noisy data, and the learned function is constituted by a decision tree. To increase human readability, the trees can be exhibited as a set of if-then rules [49]. The anatomy of a decision tree is related to a tree with the right subtree, left subtree, and the root node. The class labels are represented by the leaf nodes. Data preparation requires much less effort during pre-processing. The decision tree building process is not affected by missing values in the data. Scaling and normalization of data is not required in a decision tree. It can also handle both numerical and categorical data, even Boolean too, and normalization is not required here. It is non-parametric, and also normalization is not required in a decision tree. One major drawback of decision trees is the method of overfitting, which can be solved using the pruning method. It cannot be used in big data. It takes more time for the training time complexity to increase as the input increases. The condition on the attributes is denoted by the arcs from one node to another node.

In this, the overfitting and noise are checked by pruning the tree. The benefits of the tree-structured approach are it is easy to handle numeric and categorical attributes, interpret, and understand and it is robust with missing values [50].

### 4.7. Random Forest

A random forest classifier is an ensemble learning method that has gained tremendous interest as it is more detailed and robust regarding noise than an individual classifier and it is based on the philosophy that a set of classifiers perform better than a single classifiers does [51]. Overfitting does not happen with many features and it is very efficient in large datasets. The forest created can be reused by saving it. A random forest is a combination of classifiers with trees as base classifiers. Each classifier gives a unique vote so that the most frequent class may be assigned to the input vector (X) [52].

(26) $${\hat{C}}_{rf}^{D}=votemajority{\left\{{\hat{C}}_{d}\left(X\right)\right\}}_{1}^{D}$$

Here, votemajority {} refers to the majority of votes by each classifier for the class, and ${\hat{C}}_{d}\left(X\right)$ refers to the category forecast of the $d_{th}$ random forest tree. While training the classifiers some data may be used more than once, while some might never be used. Thus, higher classifier firmness is attained, as it makes it stronger and the classifier accuracy is improved. For designing the decision tree attribute selection metric a pruning technique is required [53]. The selection of attributes used for the decision tree has many ways, allocates a quality amount right to the attribute. The most frequent attributes are the Gini index and the information gain ratio. The random forest classifier uses the Gini index, which computes the sum of an attribute with respect to the categories. The Gini index [54] can be described in Equation (27).

(27) $$\sum \sum_{j\neq i}\left(f\left(G_{i},T\right)/\left|T\right|\right)\left(f\left(G_{j},T\right)/\left|T\right|\right)$$

where $\left(f\left(G_{i},T\right)/\left|T\right|\right)$ refers to the probability that the chosen case belongs to the category $G_{i}$. The main advantage of a random forest is that it can be used for both classification and regression problems and works well with categorical and continuous variables. It also automatically handles missing values and outliers. However, a long training period is required and it is complex in nature.

### 4.8. Logistic Regression-Stochastic Gradient Descent Classifier

Logistic Regression-Stochastic Gradient Descent (LR-SGD) is a kind of linear model, also known as incremental gradient Descent. A LR-SGD classifier is a constructive way to discriminate learning of linear classifiers under numerous penalties and loss functions such as a SVM and logistic regression [55]. The ‘log’ loss function optimizes the logistic regression while the ‘hinge’ loss function optimizes the support vector machine. The broad and sparse problems encountered in sentimental analysis make use of the LR-SGD, and this factor inspired us to use the LR-SGD in this paper. Hyperparameter tuning is a major robustness of LR-SGD which is used for resolving the error function also known as the cost function. Logistic regression [56,57] has a likelihood parameter which is expressed in Equation (28). It is computationally fast, as only one sample is processed at a time. It also converges faster for larger datasets and is easier to fit in the memory due to single training. Some drawbacks of this classifier are that it loses the advantage of vectorized operations as it deals with only a single example at a time. Secondly, due to noisy steps, it may take longer to achieve convergence.

(28) $$L\left(\theta \right)=\prod_{i=1}^{M}{\left(h_{\theta }\left(x_{i}\right)\right)}^{y_{i}}{\left(1-h_{\theta }\left(x_{i}\right)\right)}^{1-y_{i}}$$

where *M* denotes the data samples number. The following likelihood function is to be maximized to find the optimal model parameter θ.

(29) $$l\left(\theta \right)=\log\left(L\left(\theta \right)\right)=\sum_{i=1}^{M}y_{i}\log(h_{\theta }\left(x_{i}\right)+\left(1-y_{i}\right)(1-\log\left(h_{\theta }\left(x_{i}\right)\right)$$

The parameter θ can be optimized using the stochastic gradient descent classifier technique. Therefore, parameter θ can be given using Equation (30).

(30) $$\theta_{b}=\theta_{b}+\alpha \left(y_{i}-h_{\theta }\left(x_{i}\right)\right)x_{i,b}$$

where $x_{i,0}$ = 1 for all *i*.

### 4.9. Majority Voting Classifier

In this paper, the Majority Voting Classifier (MVC) has been adopted to get the best result. It is based on combining various single classifiers to obtain a highly accurate classifier from less accurate ones [58]. The combination can rectify the errors made by single classifiers on various input spaces, thereby improving the accuracy of the single classifiers used in isolation [59]. In majority voting, the forecasted outcome category is the category with the largest vote majority, i.e., the category which is the output for more than half of classifiers. It relies on the performance of many models and is not hindered by large errors from one model. It performs well in classification and regression problems. However, this classifier is more computationally intensive and thus very costly in terms of training and deploying.

(31) $$\hat{y}=mode\left\{C_{1}\left(x\right),C_{2}\left(x\right),\ldots ,C_{n}\left(x\right)\right\}$$

## 5. Results

**Figure 7:**
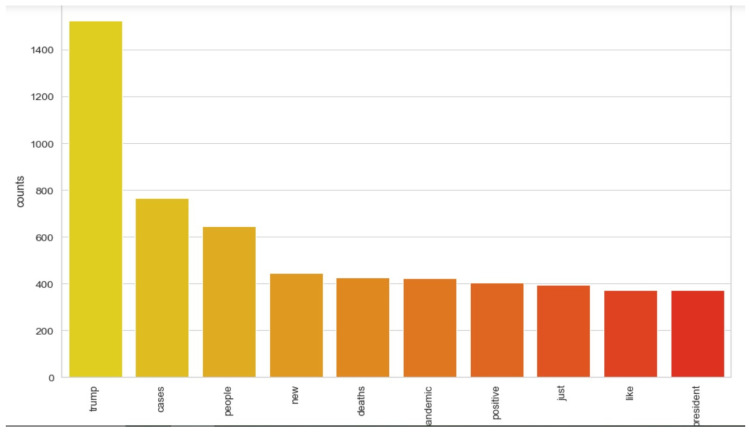
Top 10 most frequent words for the mined dataset.

**Figure 8:**
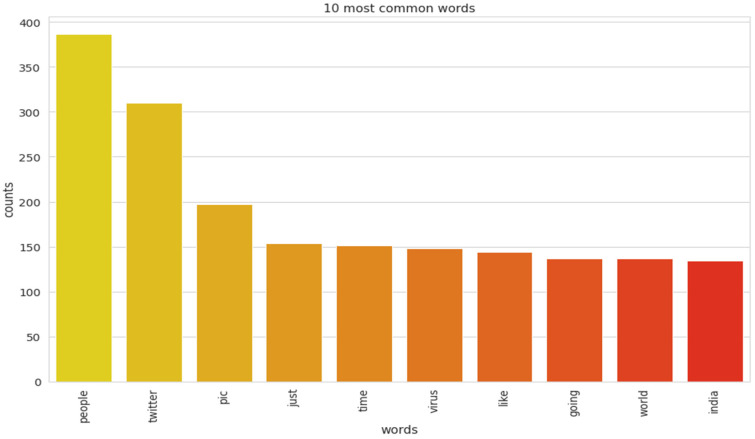
Top 10 most frequent words for the second dataset.

**Figure 9:**
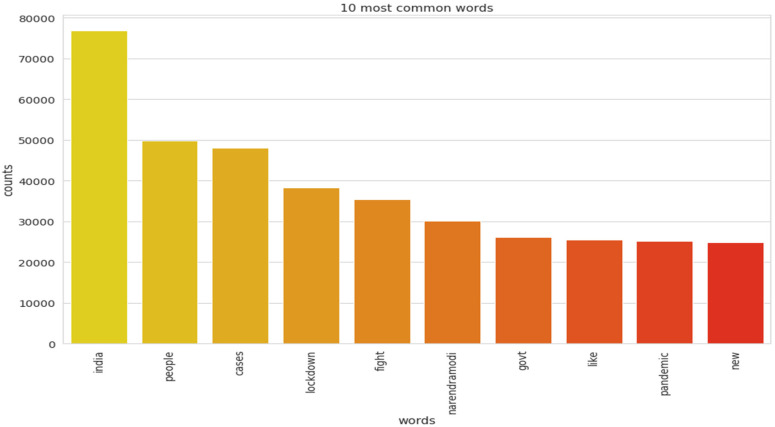
Top 10 most frequent words for the first dataset.

**Figure 10:**
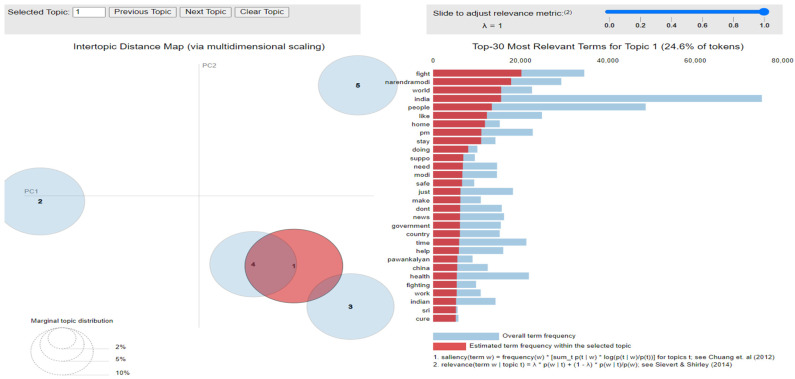
Inter-topic distance map for first dataset.

**Figure 11:**
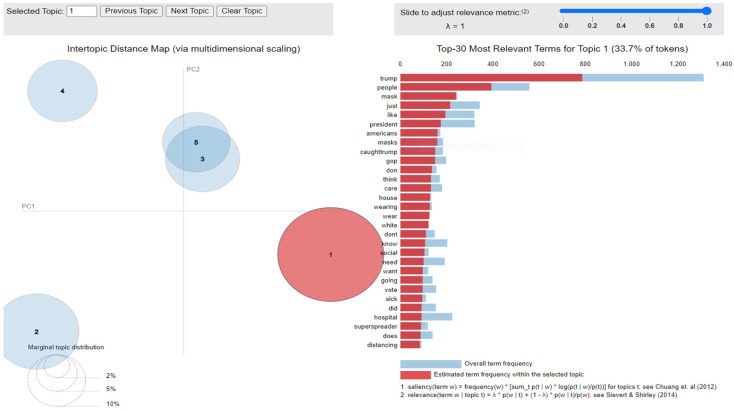
Inter-topic distance map for the mined dataset.

**Figure 12:**
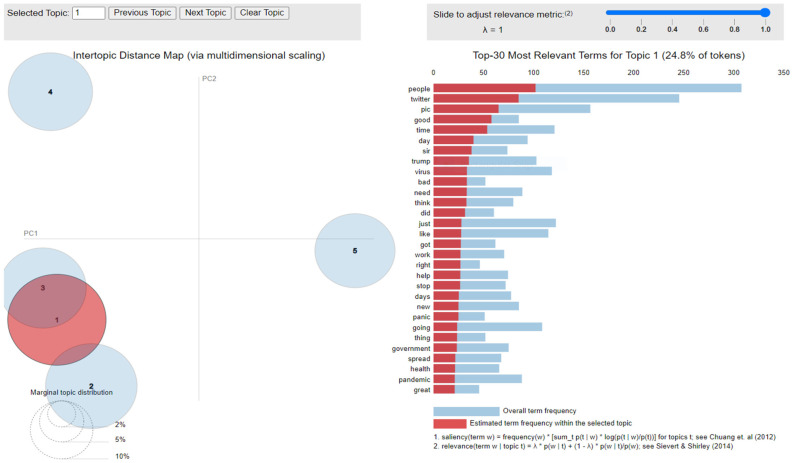
Inter-topic distance map for the second dataset.

The implementation of latent Dirichlet allocation [35] gave us a fascinating theme which makes good sense to a great extent. Before applying Latent Dirichlet Allocation (LDA) it is the principal step to analyze the text corpora, so a bar graph showing the top ten frequent words of all the datasets was plotted, as shown in Figure 7, Figure 8 and Figure 9 LDA was applied on all three datasets to detect five themes and displayed the top 10 most notable words, and the results obtained are listed in Table 2, Table 3 and Table 4. Relevance [60] and saliency [61] were introduced, which can be defined as,

(32) $$saliency\left(S\right)=f\left(S\right)\sum_{D}P\left(D/S\right)log\frac{P\left(D/S\right)}{P\left(D\right)}$$

(33) $$relevance\left(S/D\right)=\lambda P\left(S/D\right)+\left(1-\lambda \right)\frac{P\left(S/D\right)}{P\left(S\right)}$$

where S mentions a term from vocabulary data, *D* indicates a topic from the set of themes, *P*(A) is the probability of event A, and λ refers to a weight variable (0 < λ < 1). Chung et al. (2012) [61] proposed a metric saliency, which aids rapid disambiguation and classification of topics, while Sievert et al. (2014) [60] proposed a metric relevance, which bestows users with an understanding of the importance of the word in describing the topic. LDA is a technique for visualizing inter-topic depth through complex scaling forecasted on principal component axes PC1 and PC2 between the 5 themes [60]. The ranking of the top 30 most relevant and salient words in any chosen topic with λ = 1 for all the three datasets is shown in Figure 10, Figure 11 and Figure 12.

**Figure 13:**
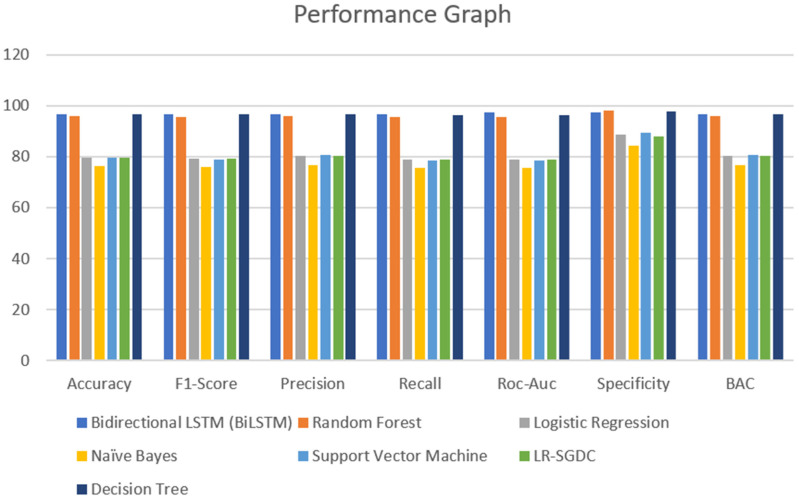
Performance graph of different classifiers for first dataset.

**Figure 14:**
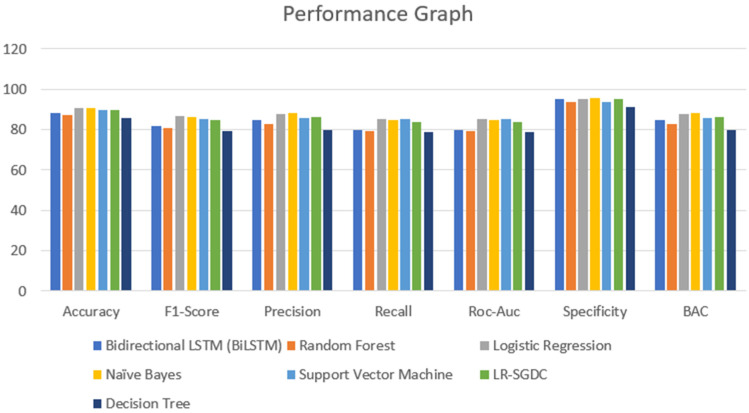
Performance graph of different classifiers for the second dataset.

A major part of the model is to evaluate it while observing the exactness and performance of classifiers on the test data and comparing the best from them. The confusion matrix [62] contains four outcomes produced by binary classifiers which can be used for describing the performance of the models. Various metrics such as recall accuracy [63], precision, AUC score, specificity, F1-score [64], and BAC were examined to verify and validate the results. The four outcomes of the confusion metric, i.e., false negative, true negative, false positive, and true positive, of various classifiers of the first and second datasets are shown in Table 5. The various evaluating metrics are shown in Table 6 and Table 7, respectively. The results of the classifiers with respect to the AUC score, F1-score, recall, accuracy, precision, BAC, and specificity are represented graphically in Figure 13 and Figure 14. The evaluating metrics are mathematically described in Equations (34)–(40).

(34) $$Accuracy=\frac{TP+TN}{TP+TN+FP+FN}$$

(35) $$Precision=\frac{TP}{TP+FP}$$

(36) $$Recall=\frac{TP}{TP+FN}$$

(37) $$F1\;score=2\times \frac{P\times R}{P+R}$$

(38) $$Specificity=\frac{TN}{TN+FP}$$

In this research paper, a Balanced Accuracy (BAC) metric has been used. BAC is calculated for an imbalanced dataset and model accuracy is represented better. It is the average of recall secured from both classes. The balanced accuracy can be calculated by using Equation (39).

(39) $$BAC=\frac{\left[\frac{TP}{TP+FP}+\frac{TN}{TN+FN}\right]}{2}$$

where *FP* is the false positive, *TN* refers to the true negative, *FN* means false negative, *TP* refers to true positive, *P* refers to precision, and *R* is the recall.

(40) $$AUC=\frac{SP-PE\left(NO+1\right)/2}{PEȗNO}$$

**Figure 15:**
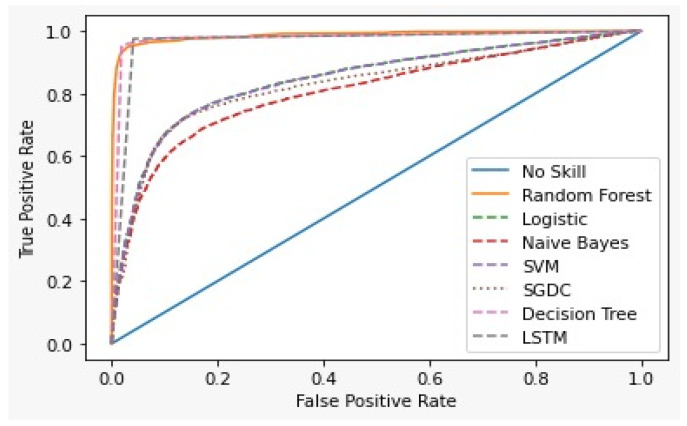
ROC curve for First dataset.

**Figure 16:**
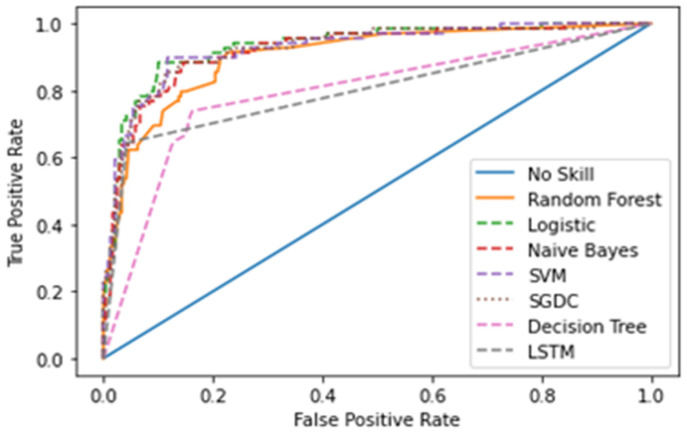
ROC curve for the second dataset.

The Receiver Operating Characteristics Curve (ROC) [65] is a graphical plot that demonstrates the characteristics ability of a binary classifier. The correlation of the False Positive Rate (FPR) and True Positive Rate (TPR) is shown using the ROC curve. It is a remarkable metric, as the entire area between 0 and 1 is covered by it. At this point, a 0.5 false positive rate is equal to a true positive rate and therefore represents a non-skilled or random classifier. The area below the ROC curve gives the AUC score. Figure 15 shows the ROC curve for the first dataset and Figure 16 for the second dataset of all the models.

In this paper, a noble dataset has been proposed. The dataset was mined from Twitter using the keyword “COVID-19”. By comparing the two datasets, i.e., the first and second, we are labelling the mined tweets. It has been seen that the first dataset gave more accurate labels than the second dataset. Table 8 shows the prediction of tweets from the first dataset and also the prediction by the authors. Table 9 shows the prediction of tweets from the second dataset and also the predictions. Table 10 and Table 11 show the number of correct and incorrect predictions by all classifiers.

Latent Dirichlet Allocation (LDA), a topic modeling technique, was applied on all three datasets related to the tweets on the COVID-19 pandemic. This led to various kinds of reactions in which the model attempted to represent a set of themes and the most appropriate words pertaining to the topic. The first dataset indicates that “India”, “people”, “cases”, “lockdown”, etc. are the most frequent topics showing that the users are very much conscious about their country and its citizens, while the second dataset emphasizes “people”, “twitter”, etc. The mined tweets have the top three topics as “Trump”, “people”, and “cases” showing that people are very much aware of COVID-19 and that most of the tweets involved the former president of the USA—this is not surprising since a majority of the users of Twitter are based in the USA. Different topics have been plotted as circles and the centers of each topic were calculated by evaluating the distance among topics. In Figure 10, Figure 11 and Figure 12, it can be seen that many topics are very close to each other and intersect each other in a few cases, thereby showing that they have many common words.

Table 6 and Table 7 show the results of the two datasets on various classifiers. It can be seen that the Bidirectional Long Short-Term Memory (BiLSTM) model performed very well on the first dataset in comparison to other classifiers with an accuracy of 96.7% and an insignificant difference between positive and negative tweets. On the other hand, logistic regression achieved a significant metric of 90.93 % accuracy on the second dataset with a large difference between the positive and negative tweets. A ROC curve for various classifiers was plotted which depicts that the BiLSTM model has the maximum area and thereby the best model for the first dataset and logistic regression for the second dataset, as shown in Figure 15 and Figure 16.

To label the mined tweets, it was very important to find the best classifier for the mined dataset so that all the classifiers were trained on the first and second datasets for predicting the results of the mined tweets. In this paper, 15 samples of the mined tweets and their predictions on various classifiers trained on both datasets are tabulated in Table 8 and Table 9. A Majority Voting Classifier (MVC) was also utilized for choosing the best classifier. We compared our predictions of the tweets with the predictions of classifiers and enumerated the number of correct and incorrect predictions and then calculated the accuracy of each classifier trained on both datasets, as shown in Table 10 and Table 11, respectively. By observing the accuracy, it was noted that the logistic regression classifier trained on the second dataset has an accuracy of 86.67%.

## 6. Discussion and Analysis

In this section, we analyze the results obtained during the experiment. Considering the results of the classifiers for the first dataset, accuracy is varied from 96.7% to 76.5%. From Table 6 and Figure 13, it can be seen that BiLSTM, random forest, and decision tree classifier models performed exceptionally well in terms of accuracy when compared with other models used for the same dataset. However, when we come to the other dataset, there is not much of a difference in the model’s accuracy which was visible in the other dataset. One of the reasons that can justify the results of the BiLSTM model is that of the use of a deep neural approach. This model has two LSTM architectures which permit the neural networks and allow both backward and forward information at every step. From this, every new result is generated from the previous instances. Coming to the other models, random forest and decision tree, both use more or less similar techniques for classifying the data points. However, in a random forest, a group of decision trees is used to provide the best results for all the trees. Due to this, in the dataset that we have used, random forest and decision tree provide promising results when compared to other models. It can be justified from Figure 14 as well.

However, when we consider the same models and expect the same results on a smaller dataset, the results are not the same. From Table 7, we tested every model on a smaller dataset and compared them with the previous result metrics. The accuracies achieved via logistic regression, naïve Bayes, SVM, and LR-SGDC were 90.93%, 90.93%, 89.96%, and 89.96%, respectively. Although these models are known for their accurate results, when it comes to the size of the dataset that considered and the relationship/dependencies among the features and target variables, these models lack in terms of accuracy. It can be justified by considering Table 6 and Table 7, from which we can see that the models that performed poorly in terms of accuracy performed well when the size of the dataset was reduced. However, if we compare the results, we find that there is not much of a difference, and the mean accuracy achieved for the second dataset is 89.03% and for the first dataset it is 86.41%.

Furthermore, looking at other result parameters, precision is considered to be a more dominating result matrix over other parameters. This is because it states the correct number of outcomes presented by the model. However, in the medical industry-based models, recall is considered to be a more efficient matrix apart from accuracy as it points out the total number of false detections given by the model. Considering our models, the mean precision value for the first dataset is 86.81%. This means that our models were able to correctly classify 86 data samples out of 100, and only 14 tweets were misclassified by the model. Looking at the other dataset with a lesser number of data samples, the mean precision value was 84.98%, which implies approximately equal results when compared to the other group of results. This could be due to the size of the data samples that were considered in the experiment. Another possible explanation could be the internal relationships that are formed by the model for classifying the results. For instance, the logistic regression model assumes a linear relationship among the data points, and based on the equations formed, performs the classification. Similarly, other models also have an internal equation based on the relationships formed, which helps in determining the results.

Similarly, recall is one of the parameters which gives the negative count of the classified samples. This parameter is also termed sensitivity. From Table 6, the average recall value is 85.79%, which is the ratio of correct positive predictions to the total number of positive data samples. Likewise, for Table 7, the mean recall value was found to be 83.93%. Apart from these four result parameters, the F1-score is among the most widely used parameter as it provides the combined detail of recall and precision. The F1-score mathematically is the harmonic mean between the precision and recall values. Since we have talked in detail about the individual parameters, the F1-score is omitted in our discussion, but for performance analysis, it can be found to be a more promising metric over individual comparison.

Another parameter that is taken into consideration apart from the performance criteria is the time complexity of the model. For this, we provide the CPU utilization time for each model that can help in providing a better viewpoint for the model selection decision. Table 12 demonstrates the time complexities for each model belonging to each dataset. For the BiLSTM model, the training time is found to be the maximum among all the classifier models; however, the average epoch training time was found to be 3141.4 and 13.2 s for the first and second datasets, respectively.

## 7. Conclusions and Future Work

In this paper, the Twitter users’ sentiments and discussions related to COVID-19 have been conveyed. The findings obtained are used to understand public sentiment and discussion of the outbreak of COVID-19 in a real-time and rapid way, aiding surveillance systems to grasp the evolving conditions. The recognized patterns and response of public tweets could be used to guide the targeted intervention strategies. Different deep learning and machine learning approaches were used for analyzing tweets. The tweets were filtered in the pre-processing part by eliminating the numbers, stopwords, URL, and various Twitter-related features with the assistance of NLTK. The features were extracted using a bag-of words model and tokenization and padding. Two datasets were used for classifying the tweets into positive or negative sentiments using different classifiers such as naïve Bayes, random forest, decision tree, SVM, logistics regression, LR-SGD classifier, bidirectional LSTM and majority voting classifier (MVC). The most suitable classifier was selected by comparing various evaluation metrics and a ROC curve. This research could be very helpful in understanding the sentiments of people in this coronavirus pandemic and could also help to avoid the fear among people by filtering out the negative comments. The government can take fruitful decisions based on the result of our application and thus reduce the chaos in the society. Through the LDA approach we can also filter out the types of tweets which can create negativity in the society. Though our approach is little bit time consuming in large datasets or high-dimensional datasets, it could be very beneficial for the society.

In this paper, a novel dataset consisting of 6648 tweets has been proposed. The dataset was mined from Twitter using the keyword “COVID-19”. We took a few tweets and labeled them to compare the results achieved by different models trained on the other two datasets. This dataset can be used for further research related to COVID-19 by utilizing various other methods. It can be executed in web and android applications to understand public opinion and control any negative sentiments or rumors related to COVID-19 in the future. This approach can also be applied on other social networking sites such as Facebook, LinkedIn, etc. to know the sentiment of the people on any topic.

## Figures and Tables

**Table 1:** Summary of Dataset.

Dataset	Positive	Negative	Total
First Dataset	80,844	96,702	177,456
Second Dataset	727	2363	3090

**Table 2:** Most prominent words using LDA in our mined datasets.

Topics	Top 10 Most Prominent Words
1	cases, trump, deaths, new, positive, total, india, active, hospital, pandemic
2	trump, people, mask, just, like, president, americans, masks, caughttrump, gop
3	pandemic, health, people, world, day, like, time, positive, test, trump
4	testing, know, test, pandemic, china, positive, days, nfl, tests, world
5	trump, news, place, good, starting, waiting, hurts, ends, jump, pockets

**Table 3:** Most prominent words using LDA for first dataset.

Topics	Top 10 Most Prominent Words
1	india, local, lets, pm, narendramodi, lockdown, app, month, buy, time
2	fight, narendramodi, world, india, people, like, home, pm, stay, doing
3	cases, india, new, positive, deaths, total, people, delhi, number, tested
4	lockdown, people, pandemic, shri, follow, india, fight, rs, food, time
5	lockdown, govt, india, sir, pmoindia, just, like, people, health, pandemic

**Table 4:** Most prominent words using LDA for second dataset.

Topics	Top 10 Most Prominent Words
1	twitter pic wajid away music suffering khan world sajid people
2	people twitter pic good time day sir trump virus bad
3	people just cases government virus going like need help days
4	people twitter india shit world cases pic like virus death
5	people home trump govt stay india going time work safe

**Table 5:** Confusion matrix of different classifiers for both the datasets.

	First Dataset				Second Dataset			
	TP	TN	FP	FN	TP	TN	FP	FN
Bidirectional LSTM (BiLSTM)	7769	9401	244	341	44	229	12	24
Logistic Regression	5509	8664	1108	2474	52	229	11	17
Random Forest	7420	9596	176	563	45	225	15	24
Naïve Bayes	5339	8244	1528	2644	51	230	10	18
Support Vector Machine	5408	8737	1035	2575	53	225	15	16
LR-SGDC	5568	8602	1170	2415	50	228	12	19
Decision tree	7597	9555	217	386	46	219	21	23

**Table 6:** The performance measure of various classifiers of the first dataset.

	Accuracy (%)	F1-Score (%)	Precision (%)	Recall (%)	Roc-Auc (%)	Specificity (%)	BAC (%)
Bidirectional LSTM (BiLSTM)	96.7	96.67	96.72	96.63	97.47	97.47	96.72
Random Forest	95.83	95.77	96.07	95.57	95.57	98.19	96.07
Logistic Regression	79.82	79.16	80.52	78.83	78.83	88.67	80.52
Naïve Bayes	76.5	75.85	76.73	75.62	75.62	84.36	76.73
Support Vector Machine	79.67	78.92	80.58	78.57	78.57	89.41	80.58
LR-SGDC	79.81	79.2	80.35	78.87	78.87	88.02	80.35
Decision Tree	96.6	96.56	96.67	96.47	96.47	97.78	96.67

**Table 7:** The performance measure of various classifiers of the second dataset.

	Accuracy (%)	F1-Score (%)	Precision (%)	Recall (%)	Roc-Auc (%)	Specificity (%)	BAC (%)
Bidirectional LSTM (BiLSTM)	88.34	81.84	84.54	79.86	79.86	95.02	84.54
Logistic Regression	90.93	86.51	87.81	85.38	85.38	95.41	87.81
Random Forest	87.37	80.89	82.68	79.48	79.48	93.75	82.68
Naïve Bayes	90.93	86.36	88.17	84.87	84.87	95.83	88.17
Support Vector Machine	89.96	85.46	85.65	85.28	85.28	93.75	85.65
LR-SGDC	89.96	84.98	86.47	83.73	83.73	95	86.47
Decision Tree	85.76	79.25	79.57	78.95	78.95	91.25	79.57

**Table 8:** Prediction of mined tweets by the various classifiers trained with first dataset.

Sl. No.	Text	First Dataset								Self
		LSTM	Random Forest	Logistic Regression	Naïve Bayes	LR-SGDC	SVM	Decision Tree	MVC	
1	#COVID-19 death toll breaches 900-mark in #Odisha	0	0	0	0	0	0	0	0	0
2	as per this data by @MoHFW_INDIA #Kerala is at the bottom in recovery rate, and top in active cases (%). Good thing is the death rate is also the lowest. State reported 3rd most cases yesterday behind #Maharashtra & #Karnataka. #COVID #COVID-19 #CoronaVirusUpdates	1	1	1	1	1	1	1	1	0
3	People working in Tech are lucky in the current situation. If they work in a big-budget company, they have moderate (only moderate) amount of job security. Counterparts in other sectors, not lucky More to be expected from COVID-19? #COVID-19 #CoronaVirus #technology #jobs	1	0	0	1	0	0	0	0	1
4	White House Aids release photos of President #Trump working while being treated for #COVID-19 #COVIDCaughtTrump	0	0	0	1	0	0	1	0	1
5	@GovMikeDeWine @LtGovHusted Where is the Prayer Day for all 209,000+ #COVID-19 DEAD AMERICANS???	0	0	0	1	0	0	0	0	0
6	Greek doctors stage 2 km fun run to debunk #COVID-19 mask myth	1	0	0	0	0	0	0	0	1
7	#LoveIsNotTourism #LoveIsEssential #COVID-19 #travelban #poetry Credit: @igneusT	0	0	0	0	0	0	0	0	0
8	Deputy Director of Narcotics Control Bureau, KPS Malhotra tests positive for #COVID-19.	1	0	0	1	0	0	1	0	0
9	All are concerned about their future. We are also concerned about when will our colleges and universities been opened up? #BREAKING #India #COVID-19 #education #reopencollege #students @EduMinOfIndia #reopen_ug_college #Health #healthcare #Trending	0	1	1	1	1	1	0	1	0
10	When a Country have a Govt who are full of #COVIDIOTS that preach Cow Urine will save one from #COVID-19	1	1	1	1	1	1	0	1	0
11	Did COVID-19 positive Donald Trump continue meeting people and attending events even after test results? #DonaldTrump #COVID-19 #coronavirus @realDonaldTrump	1	1	1	1	1	1	1	1	1
12	So basically, one man in the entire world ate one single bat, he died, and then everybody followed… I believe that’s what the scientists call #COVID-19… That’s #wild.	1	0	1	1	1	1	1	1	0
13	After 5 months of heavy usage, this 20 baht do it ourselves, still function, there also are YouTube Thai instruction mask making, M of Interior sponsored workshop in every district, we may not be top industrial countries in the world, but we find our #COVID-19 solution #Thailand	1	1	1	1	1	1	1	1	1
14	To be honest there is nothing funny about anyone over the age of 70 getting #COVID-19 and it shows the level of inhumanity from such tolerant and welcoming people who seem to be on the wrong side of overthrowing fascism!!!! Stop telling us what fascism is and stop being IT!!!”!	0	0	0	0	0	0	1	0	0
15	#Odisha: 15 more patients succumb to #COVID-19, death toll crosses 900-mark	1	0	0	0	0	0	0	0	0

Where # stands for twitter specific features (Hashtag).

**Table 9:** Prediction of mined tweets by the various classifiers trained with the second dataset.

Sl. No.	Text	Second Dataset								Self
		LSTM	Random Forest	Logistic Regression	Naïve Bayes	LR-SGDC	SVM	Decision Tree	MVC	
1	#COVID-19 death toll breaches 900-mark in #Odisha	0	1	0	0	0	0	0	0	0
2	as per this data by @MoHFW_INDIA #Kerala is at the bottom in recovery rate, and top in active cases (%). Good thing is the death rate is also the lowest. State reported 3rd most cases yesterday behind #Maharashtra & #Karnataka. #COVID #COVID-19 #CoronaVirusUpdates	0	0	0	0	0	0	0	0	0
3	People working in Tech are lucky in the current situation. If they work in a big-budget company, they have moderate (only moderate) amount of job security. Counterparts in other sectors, not lucky More to be expected from COVID-19? #COVID-19 #CoronaVirus #technology #jobs	0	1	1	1	1	1	0	1	1
4	White House Aids release photos of President #Trump working while being treated for #COVID-19 #COVIDCaughtTrump	0	1	1	1	1	1	1	1	1
5	@GovMikeDeWine @LtGovHusted Where is the Prayer Day for all 209,000+ #COVID-19 DEAD AMERICANS ???	0	0	0	0	0	0	0	0	0
6	Greek doctors stage 2 km fun run to debunk #COVID-19 mask myth	1	1	1	1	1	1	1	1	1
7	#LoveIsNotTourism #LoveIsEssential #COVID-19 #travelban #poetry Credit: @igneusT	0	1	0	0	1	1	0	0	0
8	Deputy Director of Narcotics Control Bureau, KPS Malhotra tests positive for #COVID-19.	0	1	0	1	0	0	1	0	0
9	All are concerned about their future. We are also concerned about when will our colleges and universities been opened up? #BREAKING #India #COVID-19 #education #reopencollege #students @EduMinOfIndia #reopen_ug_college #Health #healthcare #Trending	0	0	0	0	1	1	0	0	0
10	When a Country have a Govt who are full of #COVIDIOTS that preach Cow Urine will save one from #COVID-19	0	1	0	0	0	0	1	0	0
11	Did COVID-19 positive Donald Trump continue meeting people and attending events even after test results? #DonaldTrump #COVID-19 #coronavirus @ realDonaldTrump	0	0	0	0	0	0	1	0	1
12	So basically, one man in the entire world ate one single bat, he died, and then everybody followed… I believe that’s what the scientists call #COVID-19… That’s #wild.	0	1	1	1	1	1	0	1	0
13	After 5 months of heavy usage, this 20 baht do it ourselves, still function, there also are YouTube Thai instruction mask making, M of Interior sponsored workshop in every district, we may not be top industrial countries in the world, but we find our #COVID-19 solution #Thailand	0	1	1	1	1	1	1	1	1
14	To be honest there is nothing funny about anyone over the age of 70 getting #COVID-19 and it shows the level of inhumanity from such tolerant and welcoming people who seem to be on the wrong side of overthrowing fascism!!!! Stop telling us what fascism is and stop being IT!!!”!	0	1	0	1	0	0	1	0	0
15	#Odisha: 15 more patients succumb to #COVID-19, death toll crosses 900-mark	0	1	0	0	0	0	0	0	0

Where # stands for twitter specific features (Hashtag).

**Table 10:** Prediction by first dataset.

Classifier	No of Correct Prediction	No of Incorrect Prediction	Accuracy (%)
BiLSTM	9	6	60
Random Forest	9	6	60
Logistic Regression	8	7	53.34
Naïve Bayes	8	7	53.34
LR-SGDC	8	7	53.34
SVM	8	7	53.34
Decision Tree	9	6	60
Majority Voting Classifier	8	7	53.34

**Table 11:** Prediction by second dataset.

Classifier	No of Correct Prediction	No of Incorrect Prediction	Accuracy (%)
BiLSTM	11	4	73.34
Random Forest	7	8	46.67
Logistic Regression	13	2	86.67
Naïve Bayes	11	4	73.34
LR-SGDC	11	4	73.34
SVM	11	4	73.34
Decision Tree	11	4	73.34
Majority Voting Classifier	13	2	86.67

**Table 12:** Time complexity of different models for each dataset (seconds).

Classifier Model	First Dataset	Second Dataset
Random Forest	44.068	0.253
Logistic Regression	28.389	0.282
Naïve Bayes	2.682	0.035
SVM	35.448	0.069
LR SGDC	8.089	0.277
Decision Tree	206.455	0.552
BiLSTM	15,707	0.66

## Data Availability

This study consists of three datasets where dataset 1 and dataset 2 were collected from Kaggle, and for dataset 3, a GitHub link is provided as it is our own mined proposed dataset. However, for more reference, links for all the datasets are given below. Dataset 1 Link: https://www.kaggle.com/abhaydhiman/covid19-sentiments (accessed on 7 January 2022). Dataset 2 Link: https://www.kaggle.com/surajkum1198/twitterdata (accessed on 7 January 2022). Dataset 3 Link: https://github.com/satish-1999/Covid-Sentiment-Analysis/blob/main/mined_Logistic_small.csv (accessed on 7 February 2022).
